# Alternative Splicing of a Multi-Drug Transporter from *Pseudoperonospora cubensis* Generates an RXLR Effector Protein That Elicits a Rapid Cell Death

**DOI:** 10.1371/journal.pone.0034701

**Published:** 2012-04-05

**Authors:** Elizabeth A. Savory, Cheng Zou, Bishwo N. Adhikari, John P. Hamilton, C. Robin Buell, Shin-Han Shiu, Brad Day

**Affiliations:** 1 Department of Plant Pathology, Michigan State University, East Lansing, Michigan, United States of America; 2 Department of Plant Biology, Michigan State University, East Lansing, Michigan, United States of America; University of Wisconsin-Milwaukee, United States of America

## Abstract

*Pseudoperonospora cubensis*, an obligate oomycete pathogen, is the causal agent of cucurbit downy mildew, a foliar disease of global economic importance. Similar to other oomycete plant pathogens, *Ps. cubensis* has a suite of RXLR and RXLR-like effector proteins, which likely function as virulence or avirulence determinants during the course of host infection. Using *in silico* analyses, we identified 271 candidate effector proteins within the *Ps. cubensis* genome with variable RXLR motifs. In extending this analysis, we present the functional characterization of one *Ps. cubensis* effector protein, RXLR protein 1 (*Psc*RXLR1), and its closest *Phytophthora infestans* ortholog, PITG_17484, a member of the Drug/Metabolite Transporter (DMT) superfamily. To assess if such effector-non-effector pairs are common among oomycete plant pathogens, we examined the relationship(s) among putative ortholog pairs in *Ps. cubensis* and *P. infestans*. Of 271 predicted *Ps. cubensis* effector proteins, only 109 (41%) had a putative ortholog in *P. infestans* and evolutionary rate analysis of these orthologs shows that they are evolving significantly faster than most other genes. We found that *Psc*RXLR1 was up-regulated during the early stages of infection of plants, and, moreover, that heterologous expression of *Psc*RXLR1 in *Nicotiana benthamiana* elicits a rapid necrosis. More interestingly, we also demonstrate that *Psc*RXLR1 arises as a product of alternative splicing, making this the first example of an alternative splicing event in plant pathogenic oomycetes transforming a non-effector gene to a functional effector protein. Taken together, these data suggest a role for *Psc*RXLR1 in pathogenicity, and, in total, our data provide a basis for comparative analysis of candidate effector proteins and their non-effector orthologs as a means of understanding function and evolutionary history of pathogen effectors.

## Introduction

The identification and characterization of secreted effector proteins from plant pathogens has anchored the recent evolution of molecular plant pathology [1], [2], [3]. As components of many pathogenic microorganisms' secretomes, effector proteins represent a key component of phytopathogenicity, contributing to both the virulence and avirulence capacity of the invading pathogen. Numerous studies have identified and characterized the activities of secreted effector proteins from a broad range of phytopathogens [1], [4], [5]. Collectively, these works have revealed two primary functions for pathogen effector molecules. First, as virulence molecules, effector proteins can enhance a pathogen's ability to cause disease, likely through abrogating host processes that would otherwise block pathogen infection, growth, and proliferation within the host [5], [6]. Secondly, as avirulence determinants, effector proteins function to modulate the activation of host defense responses by perturbing the activity of host resistance (R) proteins [5], [6].

For infection, colonization, and subsequent propagation within their hosts to occur, pathogens must dampen multiple layers of plant defense responses. Often described as basal resistance, the initial perception and elicitation of defenses requires the recognition of pathogen associated molecular patterns (PAMPs; e.g., chitin, flagellin, LPS) [5], [6], highly conserved molecules essential for the lifestyle and survival of the microorganism. The recognition of PAMPs, which are highly specific elicitors, occurs through receptors on the host membrane surface, and following initiation of this receptor-ligand interaction, a rapid first response known as PAMP-triggered immunity (PTI) is elicited [5], [6]. Overall, the PTI response provides a basal level of resistance against a wide range of microorganisms, often utilizing conserved signaling pathways such as the up-regulation of the mitogen-activated protein kinase (MAPK) pathway, the generation of reactive oxygen species, and the induction of defense-related genes [5], [6]. To overcome PTI, phytopathogens, including bacteria and oomycetes, rely on the delivery and activity of secreted effector proteins to abrogate this initial basal level of defense, as well as to further promote virulence [3], [5], [6]. In response, pathogen effectors can be recognized by R (resistance) proteins, leading to the activation of effector-triggered immunity (ETI) [5], [6] best illustrated as an amplified and sustained layer of defense. ETI is a robust response that is often associated with the activation of a specific type of programmed cell death referred to as the hypersensitive response (HR) [5], [6]. Over time, as this cycle of subversion and recognition evolves, host specificity and subsequent interactions between pathogen and host are modulated by the interplay between the activity and recognition of secreted pathogen effector molecules and their host counterparts.

Oomycetes are a phylogenetically distinct eukaryotic lineage within the Stramenopiles, which as a group, are among the best-studied and most economically important plant pathogens. In recent years, the genomes of several agriculturally important oomycete pathogens have been sequenced, including *Phytophthora infestans*, *Phytophthora ramorum*, and *Phytophthora sojae*, the causal agents of late blight of potato and tomato, sudden oak death, and soybean root rot, respectively [7], [8]. The genomes of two other oomycete pathogens, *Pythium ultimum*, which causes damping off and root rot on a wide range of hosts, and *Hyaloperonospora arabidopsidis*, a pathogen of *Arabidopsis thaliana*, have also been sequenced [9], [10]. These investigations, through the analysis of genome content and structure, have provided a wealth of information, both towards understanding the nature of the host-pathogen interaction (e.g., host specificity, virulence strategies), as well as insight into the evolution of the interaction itself. Central to the analysis of phytopathogen genomes, the identification and characterization of oomycete effector proteins has moved swiftly into the forefront in the field of plant-pathogen interactions, due in large part to the aforementioned available genomic resources. At a primary level, the identification of a highly conserved N-terminal translocation motif (i.e., RXLR; *Arg-X-Leu-Arg*, where “X” is any amino acid) demonstrated to be necessary for effector delivery into host cells, has been a seminal discovery in the field of plant-oomycete interactions [11], [12]. Similar in function to phytopathogenic bacterial effector proteins, oomycete RXLR-containing effectors have been demonstrated to suppress PTI [13], as well as to activate ETI [11], [14], [15], [16], [17], [18]. Structurally, oomycete effector proteins display a modular organization, consisting of a N-terminal signal peptide, a conserved RXLR translocation motif, followed by a variable C-terminal effector domain [3]. It is the function and activity of the variable C-terminal effector domain that drives the activity of these molecules [3], [4].

Alternative splicing (AS) of pre-mRNA drives the generation of multiple protein isoforms through assembly of different combinations of splice sites within a single gene. In total, this process represents a conserved mechanism found in eukaryotes which drives proteome complexity within organisms with a finite number of genes [19]. In oomycetes, there are few reports of intron processing [20], [21], [22], and to date, these analyses has been strictly *in silico* [20], [22], with little functional validation [21]. Costanzo et al. [21] characterized alternative processing in *P. sojae* family 5 endoglucanases revealing the generation of both coding and non-coding RNA isoforms. Additionally, based on their large-scale analysis of intronic structure and alternative splicing in *P. sojae*, Shen and colleagues [22] validated splice variants leading to premature translation termination.

*Ps. cubensis* is an obligate biotrophic oomycete pathogen of cucurbits (i.e., cucumber, melon, squash, watermelon, etc.), and is the causal agent of cucurbit downy mildew, an economically important foliar disease [23]. Capable of rapid defoliation of fields in short periods of time (i.e., <10 days), *Ps. cubensis* is the primary factor limiting cucurbit production in the United States. Despite obvious economic importance, very little is known about the genetic determinants of virulence and pathogenicity of *Ps. cubensis*, as well as the molecular-genetic basis of resistance in the cucurbits.

Similar to related oomycete pathogens of plants, *Ps. cubensis* possesses a suite of effector proteins that likely function to promote virulence and suppress host defense responses [3], [24]. Recent work by Tian et al. [24] identified and characterized a preliminary set of effector proteins from a draft genome sequence of *Ps. cubensis* obtained using 454 pyrosequencing. In brief, this set of 61 candidate effectors included a large class of variants with sequence similarity to the canonical RXLR motif found in other oomycete plant pathogens [24]. Specifically, this work characterized the function of a QXLR-containing effector, designated *Pc*QNE, which was shown to be a member of a large family of *Ps. cubensis* QXLR nuclear-localized effectors, up-regulated during infection of cucumber. Additionally, internalization of *Pc*QNE was shown to require the QXLR-EER motif, thereby establishing a basic homology to the well-characterized *Phytophthora* spp. effector proteins.

In the current study, we describe the identification and evolutionary potential of the *Ps. cubensis* effector repertoire. First, through characterization of a RXLR effector protein, *Psc*RXLR1, we investigated the localization and *in planta* activity, and similarly to some oomycete effector proteins described to date, *Psc*RXLR1 induces a rapid cell death response when delivered into plant cells. Additionally, using whole transcriptome sequencing analyses, as well as RT-PCR, we show that *Psc*RXLR1 is a product of alternative splicing of the *Psc*_781.4 gene which encodes a putative multi-drug transporter. Coupled with the induction and expression of *Psc*RXLR1 mRNA during *Ps. cubensis* infection of cucumber, as well as a complement of bioinformatic, cell biology and *in vivo* analyses, we provide evidence suggestive of a virulence role for *Psc*RXLR1. Finally, we used *Psc*RXLR1 as template for assessing the conservation and evolutionary potential of oomycete effector proteins from *Ps. cubensis*, identifying and analyzing orthologous pairs of *Ps. cubensis* effector proteins and *P. infestans* non-effector proteins. Using more robust methods, we identified additional candidate effectors from *Ps. cubensis* for these analyses and showed that, like other oomycete effectors, they tend to be influenced by positive selection. Assessment of evolutionary rate and conservation of secretion signals between orthologous pairs revealed that *Ps. cubensis* effectors are undergoing adaptive evolution and conservation of signal peptides are similar among effector and non-effector proteins in *Ps. cubensis*. Overall, our study provides support for the investigation of relationships among oomycete effectors and their non-effector orthologs, and in total, the analysis presented herein establishes a foundation for understanding the evolution of effector repertoires and host-pathogen specificity.

## Results

### Genome sequencing of *Ps. cubensis*

Next generation sequencing with the Illumina Genome Analyzer II platform was used to generate an assembly of the *Ps. cubensis* MSU-1 genome. A total of 4.5 Gb of cleaned paired end reads from two libraries were used to generate the assembly using Velvet, a *de novo* short read assembler [25]. The final assembly contains 35,546 contigs with an N50 contig size of 4.0 Kbp representing 64.4 Mbp. Protein coding genes in the draft assembly were annotated using MAKER [26] which incorporated *ab initio* gene predictions, protein evidence, and transcript evidence from other sequenced oomycete genomes. In total, 23,519 loci and 23,522 gene models were predicted.

### Identification of the *Ps. cubensis* effector repertoire

Our initial analysis of the effector complement of *Ps. cubensis* in an earlier draft assembly [24] identified 61 sequences containing the conserved RXLR, or novel QXLR, motif found in known oomycete effector proteins. This number is significantly less than the effector count predicted for other plant pathogenic oomycetes (i.e., 563 effectors in *P. infestans*, 396 in *P. sojae*, 374 in *P. ramorum*, and 134 in *H. arabidopsidis*; [7], [10], [27]), and is likely the result of limited coverage generated from an initial 454 pyrosequencing [24]. Generation of genomic sequences using the Illumina Genome Analyzer platform and their subsequent assembly generated a more comprehensive dataset. Using this resource, 269 additional sequences were identified as putative effector proteins. Interestingly, the putative *Ps. cubensis* effectors showed more variation at the R1 position of the RXLR motif than previously shown [24], with 18 amino acids predicted at the R1 position, in addition to R and Q (Table S1). Moreover, we have evidence for expression for at least one predicted effector with any one of 19 amino acids (except Y, *Tyr*) at position R1, during the course of infection on a susceptible cucumber cultivar (Table S1; Savory et al., PLoS ONE, in press), supporting the hypothesis of an expanded translocation motif repertoire in *Ps. cubensis*. In total, including the previously characterized *Pc*QNE, the current predicted effector complement of *Ps. cubensis* contains 271 members.

### Nature of selection on *Ps. cubensis* paralogs

**Figure 1:**
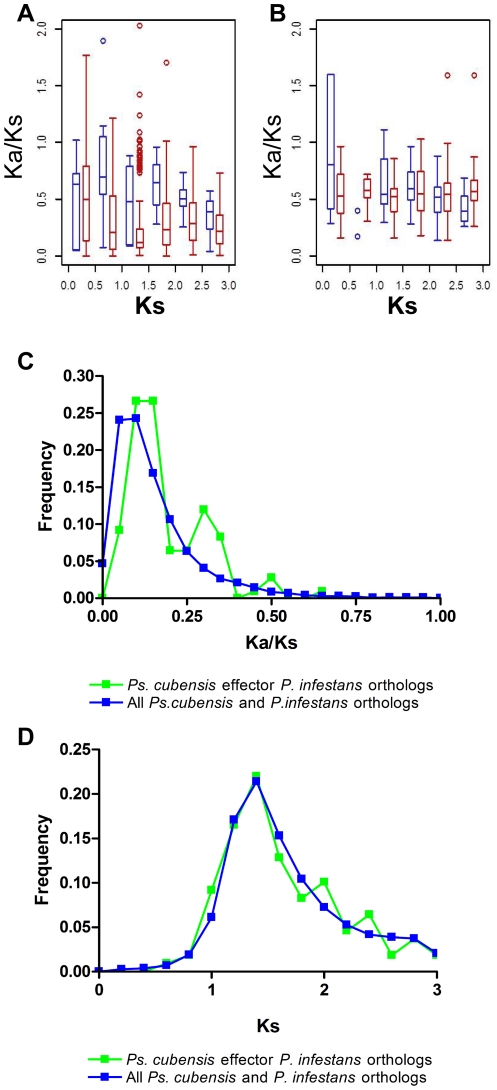
Strength of purifying selection on *Pseudoperonspora cubensis* effectors. Comparison of selective constraints on effector paralogs (blue) and all other genes (red) in (A) *Ps. cubensis* and (B) *P. infestans*. Frequency distributions (C) of *ω*, the ratio between the nonsynonymous substitution rate (*Ka*) and the synonymous substitution rate (*Ks*) of *Ps. cubensis* and *P. infestans* sequence pairs. Distributions of *Ks* values (D) of *Ps. cubensis* and *P. infestans* sequence pairs. Green symbols indicate *Ps. cubensis* effector – *P. infestans* non-effector ortholog pairs. Blue symbols represent other orthologous gene pairs between the two species.

Based on comparative genomic analyses of several oomycete plant pathogens, positive selection has been postulated to act disproportionately on effectors gene[s] compared to other genes in the genome [27], [28]. To this end, we examined the strength of selection acting upon the predicted effector complement of *Ps. cubensis* by estimating ω, the ratio of the non-synonymous substitution rate (*Ka*) to the synonymous substitution rate (*Ks*). Among all *Ps. cubensis* effector paralogs, the median ω is 0.54, which is significantly higher than that of *Ps. cubensis* paralogous genes in general (ω = 0.24, Wilcox Rank Sum Test, *p*<2.2e-16). For comparison, we also examined *P. infestans* effectors and arrived at the same conclusion (Wilcox Rank Sum Test, *p*<4.0e-14). Because more recent duplicates tend to have elevated ω, we examined if the higher ω values among effector paralogs can be attributed to recent duplication. We found that, using *Ks* as a proxy of time, the ω values for effector pairs are in general significantly higher than other paralogs in *Ps. cubensis* in a *Ks* bin (Figure 1A). Thus, the elevated ω values among effectors are not exclusively due to relaxation of selection among recent duplicates. The results for *P. infestans* effectors are similar (Figure 1B), although the ω values of *P. infestans* are in general higher than those in *Ps. cubensis*.

Taken together, *Ps. cubensis* effectors either have experienced a significantly lower degree of selective constraints, or tend to be positively selected. Consistent with the latter, 6.3% of *Ps. cubensis* effector paralog pairs have ω>1, compared to 3.2% of all other paralogous gene pairs. In parallel to our observations in *Ps. cubensis*, 4.6% of *P. infestans* effector paralog pairs have ω>1, compared to 3.2% for all other paralogs. Although there is no clear evidence suggesting that most effectors are subjected to positive selection, it is interesting that even among relatively ancient effector duplicates, the rate of evolution among effectors is significantly higher than most genes. Given that older duplicates that survive for tens to hundreds of millions of years tend to be subjected to substantially stronger selective constraints than young duplicates [29], this would suggest that, perhaps, effectors function in a way that do not require as strong a constraint on their primary sequence. Alternatively, it is also possible that pathogen effectors, even those having undergone ancient duplication events, experience some degree of continuous positive selection.

### Relationship between *Ps. cubensis* effectors and their *P. infestans* orthologs

Subsequent *in silico* analysis of candidate *Ps. cubensis* effectors and comparisons to annotated genes in *P. infestans* revealed that there were a number of orthologs between *Ps. cubensis* effector proteins and both effector and non-effector genes in *P. infestans*. We hypothesized that this scenario (i.e., effector with non-effector ortholog) may provide a foundation for the analysis of the evolution of effectors from non-effector proteins. Therefore, we identified orthologous pairs of predicted *Ps. cubensis* effector proteins and their non-effector counterparts in *P. infestans* considering sequence similarity and synteny (see Materials and Methods). With this approach, 11,601 orthologous gene pairs were identified between *Ps. cubensis* and *P. infestans* for comparison. Of 271 *Ps. cubensis* effector sequences, 109 had a predicted ortholog in *P. infestans* (Table S2). As shown in Figure 1C, the *Ps. cubensis* effector *P. infestans* (*Psc*E-*Pi*) ortholog pairs have significantly higher ω values as compared to the baseline pairs (Kolmogorov-Smirnov test, *p*<7.9e-06), consistent with what was found with the effector paralogs (Figure 1A). Additionally, the distribution of ω for the *Psc*E-*Pi* pairs appears multi-modal. Given that the first effector ortholog peak (at ω∼0.15) is mostly overlapping with that of the other orthologs, these effector paralogs are more highly conserved. The second peak at ω∼0.3 likely indicates the presence of a group of effectors that are more quickly evolving (Figure 1C). However, we cannot rule out the possibility that these peaks are present simply due to the small effector ortholog sample size.

To determine if the overall higher ω value among effector orthologs is an artifact due to mis-identification of orthologous genes, we examined if putative effectors, as well as the other orthologs, have similar “age”. As shown in Figure 1D, the distributions of *Ks* values for the effector and the other orthologs are highly similar and are statistically indistinguishable. Thus, mis-identified orthologous pairs likely do not significantly impact our findings.

### Signal peptide conservation among ortholog pairs

Signal peptides are essential components of oomycete effector proteins, as they are required for translocation of the protein from the pathogen haustorium to the extrahaustorial matrix prior to uptake by the host cell membrane [3]. As such, all 109 of the *Ps. cubensis* effector sequences in the *Psc*E-*Pi* dataset are predicted to have signal peptides (Figure S1). However, only 71 (65%) of the corresponding *P. infestans* orthologs were predicted to be secreted proteins. For comparison, predictions of signal peptides were made for 10,383 of the 11,601 ortholog pairs. Of these, there were 688 (6.63%; *Psc*-sec/*Pi*-sec) ortholog pairs where both members were predicted to have signal peptides, 428 (4.12%; *Psc*-sec/*Pi*-non) pairs where the *Ps. cubensis* protein was predicted to be secreted and the *P. infestans* ortholog was not, and 622 (5.99%; *Psc*-non/*Pi*-sec) where the *Ps. cubensis* sequence did not have a predicted signal peptide and its corresponding *P. infestans* sequence was predicted to be secreted. Additionally, there were 8,645 (83.3%; *Psc*-non/*Pi*-non) ortholog pairs where neither member was predicted to be secreted. For statistical analysis, the *Psc*-sec/*Pi*-sec and *Psc*-sec/*Pi*-non datasets from the *Ps. cubensis* effector-*P. infestans* orthologs were compared to their respective genome-wide datasets. Using the Fisher's exact test, no significant difference (p = 0.5354) was identified between the two datasets, indicating that presence of signal peptide prediction is not a good indicator of potential selection for effector peptide evolution.

### Identification of *Pseudoperonospora cubensis* effector *Psc*RXLR1

**Figure 2:**
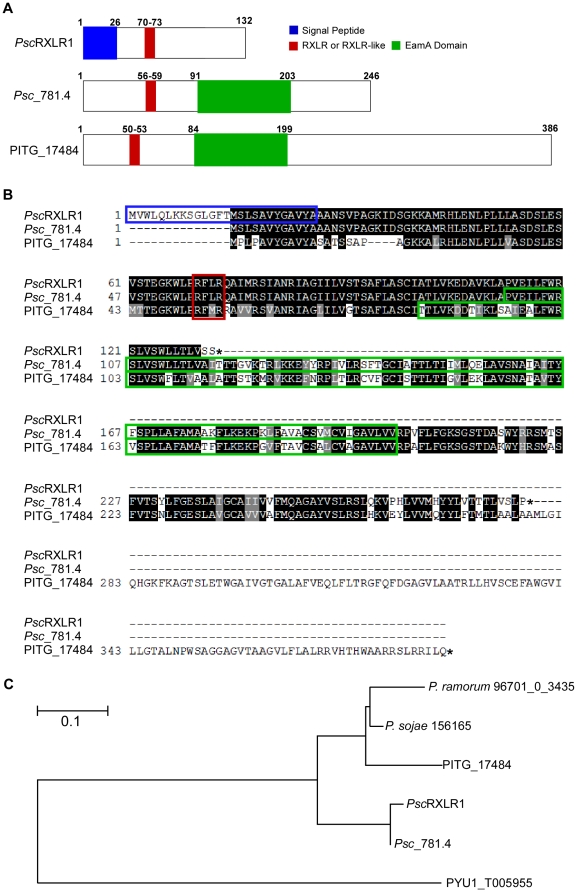
*Psc*RXLR1 encodes a RXLR-containing effector protein with homology to a non-effector protein in *Phytophthora infestans*. (A) Schematic representations of *Psc*RXLR1, *Psc*_781.4 and PITG_17484 from *P. infestans*. (B) Multiple sequence alignment of *Psc*RXLR1, *Psc*_781.4 and PITG_174984. Protein sequences were aligned using CLUSTALW and shaded for consensus using BOXSHADE. The asterisk indicates a stop codon. (C) The full length protein sequences of *Psc*RXLR1, *Psc*781.4 and their orthologs from *P. infestans* (PITG_17484), *Phytophthora ramorum* (*P.* ramorum 96701 0 3435), *Phytophthora sojae* (*P. sojae* 156165) and *Pythium ultimum* (PYU_T005955) were aligned using Muscle and evolutionary history was inferred by using the Maximum Likelihood method based on the JTT matrix-based model [1] using MEGA5 [2]. 500 bootstrap runs were performed.

Using the RXLR effector identification pipeline [28], we previously identified 61 candidate effector protein sequences from a draft genome assembly of *Ps. cubensis* [24]. Initial analysis of these sequences using the Basic Alignment Analysis Search Tool (BLAST) against the proteome of *P. infestans* revealed that only 7 of these sequences aligned with annotated proteins within the *P. infestans* genome database; moreover, only 1 of these was predicted to be a secreted RXLR effector. Of these sequences, one (contig01871_F1) had 75% amino acid identity to *P. infestans* protein PITG_17484, a putative member of the drug/metabolite transporter (DMT) superfamily (CLO184; Figure 2). Additional cloning via 3′ RACE PCR and subsequent analysis revealed that the *Ps. cubensis* coding sequence, hereafter referred to as *Ps. cubensis* RXLR protein 1 (*Psc*RXLR1), appeared significantly shorter (i.e., 132 amino acids), compared to its *P. infestans* ortholog PITG_1784 (i.e., 332 amino acids). This apparent truncation in *Psc*RXLR1 results in a protein coding sequence lacking the EamA functional domain (PF00892; formerly called DUF6) found in members of the DMT family [30].

SignalP analysis of *Psc*RxLR1 identified a putative 26 amino acid signal peptide at the N-terminus of the protein (Figure 2). Based on the conserved features and domain organization of oomycete effectors, the presence of a canonical RXLR motif was identified at amino acid position 70 (Figure 2). However, unlike several previously characterized oomycete effector proteins, *Psc*RXLR1 does not contain an EER motif, which has also been implicated in oomycete effector translocation into the host cell [12], [24], [27]. The *P. infestans* ortholog, PITG_17848, while not having a predicted signal peptide, does contain an RXLR-like motif (i.e., RFMR; Figure 2A). To eliminate the possibility that PITG_17484 was mis-annotated and did in fact contain a signal peptide upstream of the predicted coding sequence, the region 500 bp upstream of the annotated PITG_17484 sequence was examined and a canonical signal peptide sequence was not identified. We therefore concluded that PITG_17484 is not an RXLR effector protein.

The absence of a predicted signal peptide in PITG_17848 suggests that *Psc*RXLR1 may have evolved this function independently. To address this possibility, and to further explore the ancestral function of these proteins, orthologous *Psc*RXLR1 sequences in additional plant oomycete pathogen species were identified, including *P. sojae*, *P. ramorum*, and *Py. ultimum*. Not surprisingly, the sequences from *P. sojae* and *P. ramorum* were more similar to *Psc*RXLR1 than those from *Py. ultimum* (76% and 72%, respectively, compared to 59%; Figure S2). Additionally, while none of these orthologs had predicted signal peptides, they did contain EamA functional domains, indicating that they were also members of the DMT superfamily (Figure S2). Phylogenetic analysis to infer evolutionary relationships between *Psc*RXLR1 and orthologs from *P. sojae*, *P. ramorum*, and *Py. ultimum* supported these observations (Figure 2C).

### Functional validation of the *Psc*RXLR1 signal peptide

**Figure 3:**
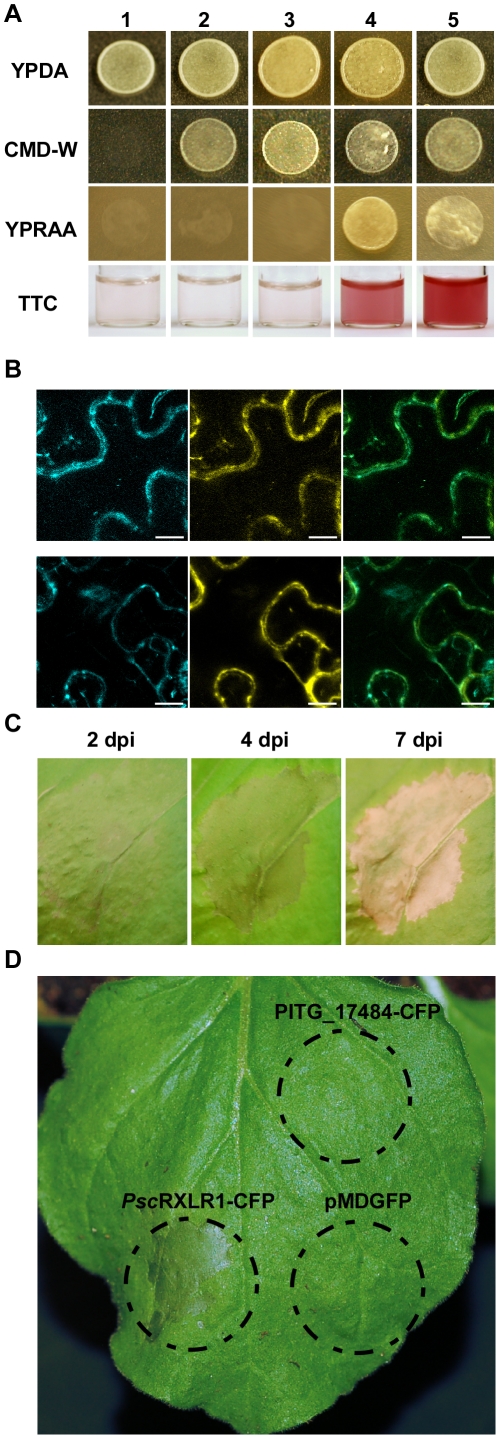
Functional characterization of *Psc*RXLR1 and PITG_17484. (A) *Psc*RXLR1 has a functional signal peptide validated by the yeast signal trap assay. Yeast strains were grown on media with raffinose (YPRAA) as the sole carbon source or in the presence of 2,3,5-triphenyltetrazolium chloride (TTC). Yeast strains YTK12 and YTK12∶pSUC2 EV both lack a functional invertase gene and cannot grow on YPRAA medium or reduce TTC to red formazan. The functional signal peptide of *Psc*RXLR1, when fused in-frame to the mature yeast invertase (pSUC2-*Psc*RXLR1), allows for secretion of invertase, resulting in growth on YPRAA medium, as well as reduction of TTC to red formazan. PITG_17484, as predicted, does not have a functional signal peptide (pSUC2-PITG_17484). (B) Both *Psc*RXLR1_CFP (top row) and PITG_17484_CFP (bottom row) co-localize with a plasma membrane-specific *At*PIP2A_YFP marker. Left panels: C-CFP fusion protein only. Center panels: *At*PIP2A_YFP. Right panels: Merge of CFP and YFP images. Scale bar = 10 µm. (C, D) Heterologous expression of *Psc*RXLR1 induces chlorosis and necrosis in *Nicotiana benthamiana*. *Agrobacterium tumefaciens* C58-C1 expressing *Psc*RXLR1_CFP, PITG_17484_CFP, or an *At*PIP2A-YFP construct were infiltrated into *N. benthamiana*. The chlorosis and necrosis phenotype of *Psc*RXLR1_CFP infiltrated leaves (C) was photographed at 2, 3, and 7 days post-inoculation (dpi). Leaf areas infiltrated with either PITG_17484_CFP or *At*PIP2A-YFP (D) as designated by the dash line circles did not shown any phenotype at 3 dpi.

A primary characteristic of oomycete effector proteins is signal peptide-mediated secretion from the haustoria into the extrahaustorial matrix (EHM) prior to translocation into the host cell [3]. *Psc*RXLR1 contains a 26 amino acid signal peptide as predicted by SignalP 3.0 ([31]; HMM Probability, 0.966), whereas its closest *P. infestans* ortholog, PITG_17484, does not have a predicted signal peptide. To determine if the predicted signal peptide from *Psc*RXLR1 is functional, we used a yeast signal trap assay based on the requirement of invertase secretion for yeast growth on media with raffinose as the sole carbon source [32]. This assay has been used previously to confirm predicted signal peptide sequences in candidate effector proteins from both *P. infestans* and *Ps. cubensis* [24], [33]. As shown in Figure 3A, pSUC2-*Psc*RXLR1 (column 4) is able to grow on medium containing only raffinose (YPRAA), indicating that the signal peptide of *Psc*RXLR1 is sufficient for secretion of invertase. As a second confirmation of signal peptide function, we monitored the reduction of 2,3,5-triphenyltetrazolium chloride (TTC) to the red-colored compound triphenylformazan. Again, pSUC2-*Psc*RXLR1 (column 4) was confirmed as having a functional signal peptide. In contrast, neither the control yeast strains (i.e., YPK12 [column 1] or pSUC2 [column 2]), nor the pSUC2-PITG_17484 (column 3) yeast strain containing a PITG_17484-invertase fusion construct, grew on YPRAA, nor were they able to reduce TTC. Our positive control, PcQNE-SP1 (5) was, as shown previously [24], both able to grow on YPRAA medium and reduce TTC. These data support the annotation of *Psc*RXLR1 as a secreted RXLR effector protein and confirm that its *P. infestans* ortholog is a non-secreted protein.

### *Psc*RXLR1 and PITG_1784 localize to the plant plasma membrane

*In planta* localization of effector proteins has been successfully used to guide functional analysis and to infer the function itself [34], [35], [36], [37]. To identify a possible role for *Psc*RXLR1 in the pathogenicity of *Ps. cubensis*, and to provide clues as to its putative role *in planta*, we investigated the localization of *Psc*RXLR1. To this end, a C-terminal CFP-fusion protein (i.e., *Psc*RXLR1-CFP) was transiently expressed in *Nicotiana benthamiana*, and protein localization visualized using laser scanning confocal microscopy. Based on the similarity of *Psc*RXLR1 with members of the DMT superfamily in *Phytophthora* spp. and *Py. ultimum*, *Psc*RXLR1 was predicted to localize to the plant plasma membrane, despite the absence of a predicted transmembrane domain when analyzed using InterProScan. To confirm this, *Psc*RXLR1-CFP was co-expressed with a YFP-tagged construct encoding the aquaporin gene *At*PIP2A, a marker for plasma membrane localization [38]. As predicted, *Psc*RXLR1-CFP co-localized with *At*PIP2A-YFP (Figure 3B), confirming that *Psc*RXLR1 localizes to the plasma membrane *in planta*. Additionally, a C-terminal CFP fusion was made to the *P. infestans* ortholog PITG_17484, which was also confirmed to be plasma membrane localized (Figure 3B).

### *Psc*RXLR1, but not its *P. infestans* ortholog, elicits a rapid cell death response when expressed in *Nicotiana benthamiana*

The obligate nature of a plant pathogen often presents challenges towards functional characterization of virulence and susceptibility within their respective host(s). To circumvent this limitation, transient heterologous systems have been developed and have proved valuable in their functional characterization [34], [39], [40], [41], [42]. To investigate the activity of *Psc*RXLR1 *in planta*, we utilized heterologous expression in *N. benthamiana* as means to characterize and determine the function of *Psc*RXLR1. As shown in Figure 3C, expression of *Psc*RxLR1 resulted in leaf chlorosis throughout the entire infiltration zone by 2 dpi, followed by browning and initiation of necrosis at 4 dpi. By 7 dpi, the zone of infiltration was completely dehydrated. In comparison, neither infiltration with PITG_17484, nor pMDGFP, resulted in any detectable cell death-type phenotype in *N. benthamiana* leaves at 4 dpi (Figure 3D; Figure S3).

### *Psc*RXLR1 expression is induced during *Ps. cubensis* infection of cucumber

**Figure 4:**
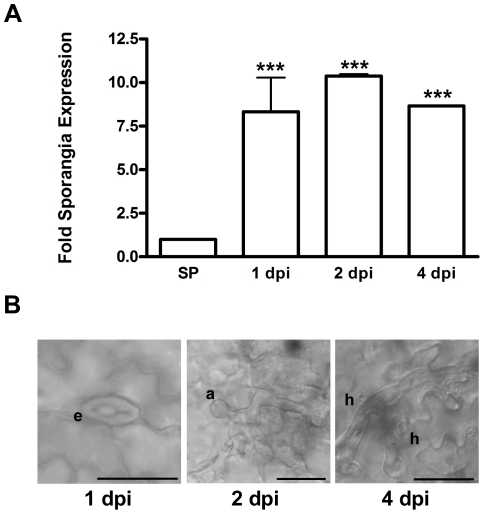
*Psc*RXLR1 mRNA expression is up-regulated during *Pseudoperonospora cubensis* infection of cucumber. (A) Expression levels of *Psc*RXLR1 in sporangia (SP) and at 1, 2, and 4 days post-inoculation (dpi). Expression is displayed as fold sporangia expression and all time points are significantly different compared to SP control (*** indicates p<0.001) using Two-way ANOVA. Error bars represent the standard error of the mean of 2 technical replicates from each of 2 biological replicates. (B) Differential interference (DIC) microscopy images of stages of *Ps. cubensis* infection on cucumber where e = encysted zoospore; a = appressorium; and h = haustorium. Scale bars = 25 µm.

The function of pathogen effector molecules is to enhance the virulence of the pathogen during its lifecycle, as well as to dampen host defense responses activated during infection. In this regard, the temporal expression of effector molecules during infection and pathogen development often signals critical stages in the host-pathogen interaction. Expression of *Psc*RXLR1 mRNA was measured using quantitative reverse transcription (qRT)-PCR following infection of *Ps. cubensis* on the susceptible cucumber cultivar ‘Vlaspik’. As shown in Figure 4, expression of *Psc*RXLR1 was significantly (p<0.001) induced during infection, beginning at 1 dpi and continuing through 4 dpi, as compared to the basal expression level in sporangia. Induction of gene expression at 1 dpi corresponds with zoospore encystment in the stomata, the first stage of pathogen entry into the host (Figure 4B, left panel). Subsequent expression observed through 4 dpi corresponds with hyphal penetration through the stomata, growth throughout the mesophyll, and formation of haustoria (Figure 4B, center and right panels). This pattern of expression supports a potential role for *Psc*RXLR1 in initial establishment of the infection possibly through dampening host defense responses. Additionally, this pattern is consistent with the expression patterns observed in other oomycete plant pathogen effectors, further supporting the prediction of *Psc*RXLR1 as an effector protein with a role in infection and disease development.

### *Psc*RXLR1 is a splice variant of *Psc*_781.4

**Figure 5:**
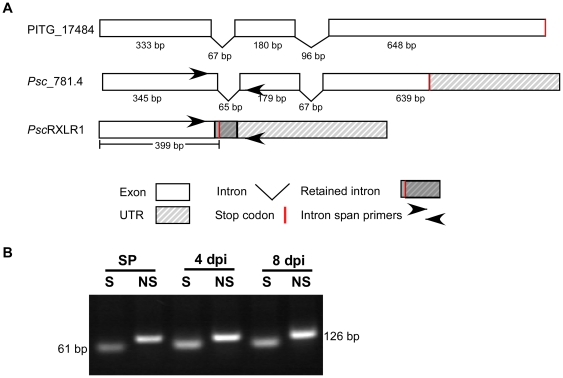
*Psc*RXLR1 is a splice variant of *Psc*_781.4. (A) Schematic representation of intron and exon structures of PITG_17484, *Psc*_781.4, and *Psc*RXLR1. (B) RT-PCR analysis of alternative splicing in *Psc*_781.4 in sporangia (SP) and at 4 and 8 days post-inoculation (dpi). RT-PCR products were subcloned into the pGEM vector and DNA was amplified by PCR using intron spanning primers as shown in (A). S, transcript with spliced intron 1. NS, non-spliced transcript.

Automated annotation of the Illumina-generated *Ps. cubensis* assembly described in this study resulted in *Psc*_781.4, a gene model at the *Psc*RXLR1 locus that more closely mirrored PITG_17484 than our prediction for *Psc*RXLR1 and what was obtained through molecular cloning (Figure 5), with the primary difference between the two predictions being that intron 1 is either spliced in *Psc*_781.4, or retained in *Psc*RXLR1 (Figure 5A). Empirical whole transcriptome sequence data (RNA-seq) from *Ps. cubensis* sporangia (unpublished results) provides support for both isoforms at this locus. When the first intron is retained, a stop codon is also brought into frame, yielding a truncated transcript (i.e., putative effector *Psc*RXLR1), and subsequently, a smaller protein, which, as described in Figure 2, lacks the EamA functional domain (Figure 5A).

Based on our *in silico* predictions we confirmed which gene model, or both, was represented *in vivo*. Using an RT-PCR-based approach, we were able to amplify both splice variants from purified sporangia (SP), as well as from infected leaf material harvested at 4 and 8 days post-inoculation (dpi) (Figure 5B), suggesting that both isoforms are present throughout the infection process. As an added control, the resultant PCR products were cloned and sequenced to confirm that they corresponded to the appropriate splice variant (Figure S4). Additional functional analysis of *Psc*_781.4 confirmed transient expression in *N. benthamiana* does not elicit a cell death response *in planta*, indicating that it likely has no virulence function in *Ps. cubensis* (Figure S5). In total, these independent methods confirm our conclusion that *Psc*_781.4 is alternatively spliced leading to generation of a functional RXLR effector protein.

## Discussion

In this study, we describe a candidate RXLR-type effector from *Ps. cubensis* that results from a splice variant of a putative multi-drug transporter protein, and additionally expands the scope of our understanding of the function and evolutionary history of the *Ps. cubensis* effector repertoire. While *Ps. cubensis* is an oomycete pathogen of worldwide economic importance, insight into the mechanism(s) underlying its virulence and pathogenicity remain limited [43]. A recent study has provided a foundation for investigating the genetic basis for virulence and pathogenicity in *Ps. cubensis* through generation of a large scale genomic dataset [24]. We build upon this previous work using a combination of *in silico* analyses, gene expression studies, and cell biology to functionally characterize *Psc*RXLR1 and establish a potential role in promoting *Ps. cubensis* infection and proliferation.

Alternative splicing has been previously described in oomycetes pathogens of plants; specifically related to the family 5 endoglucanases (EGL5) from *P. sojae* [21], as well as in gene families such as Crinklers (CRNs), protein kinases, and transcription factors [22]. In *P. sojae*, EGL5 proteins have a role in infection of soybean and are highly up-regulated during the early stages of infection. As part of these analyses, four different mechanisms of alternative splicing were described: intron skip, exon skip, alternative donor site, and alternative acceptor site, with intron skip, where the intron is retained, being the most commonly observed mechanism [22]. In agreement with this previous observation, we propose that the *Psc*_RXLR1 transcript is generated *via* a retained intron from *Psc*_781.4, yielding an RXLR-type effector. From an evolutionary standpoint, alternative-splicing functions to expand the capacity of an organism's proteome, thus enabling the generation of multiple functional isoforms from a single coding sequence. Over time, new isoforms may be maintained if they have a beneficial function [44], or lost, if their function is not critical to the lifecycle of the organism. In the case of plant pathogens, this process could potentially serve an adaptive role to allow for generation of isoforms of “housekeeping” type genes that gain new function(s), potentially to promote virulence or infection. Alternatively, the *Psc*RXLR1 splice variant could represent a step in evolutionary time, as it is generated from the same coding sequence as *Psc*_781.4 and maintained in the coding repertoire, but has not been duplicated or retained as a separate sequence.

Like other oomycete effectors characterized to date, *Psc*RXLR1 has a functional 26 amino acid signal peptide necessary for secretion from the haustorium into the extrahaustorial matrix prior to translocation into the host cytoplasm. Interestingly, *Psc*RXLR1 is also localized to the host plasma membrane, despite the lack of a predicted transmembrane domain or localization signal (Figure 3). To further examine this, we surveyed the genomes of additional oomycete plant pathogens for orthologous sequences. We hypothesize that if functional characterization data for orthologs in any of these better characterized species (e.g., *P. infestans*, *P. ramorum* and *P. sojae*) existed, it might provide insight into both the function and conservation of *Psc*RXLR1. Through BLAST analysis of the *P. infestans*, *P. sojae*, *P. ramorum* and *Py. ultimum* genomes, we identified orthologous sequences in each of the pathogens, all of which were annotated as members of the Drug/Metabolite Transporter (DMT) superfamily [30]. The DMT superfamily encompasses 19 families; the orthologs described here are members of the EamA family, named for the O-acetylserine/cysteine export gene in *Escherichia coli* [45]. While *Psc*RXLR1 is lacking the EamA transmembrane domains that are characteristic of these transporter proteins, our data clearly demonstrated plasma membrane localization.

Monitoring the expression of both pathogen and host genes during infection can provide insight into the interplay between resistance and susceptibility [46]. Using qRT-PCR, we demonstrated that expression of *Psc*RXLR1 mRNA is up-regulated during the early stages of infection of cucumber. While we were unable to distinguish between isoforms, expression was induced nearly 10-fold at 1, 2 and 4 days post-inoculation (dpi), corresponding with zoospore encystment, appressorium formation and penetration, and proliferation and haustoria establishment, respectively (Figure 5). Several effector proteins from *P. infestans* have also been demonstrated to have distinct temporal patterns of expression, and are often expressed during the pre- and early stages of infection, representative of the biotrophic phase of the *P. infestans* life cycle [12]. Based on the robust induction of *Psc*RXLR1 mRNA during the early stages of infection, as well as the aggressive nature of the necrosis-inducing activity observed in *N. benthamiana*, we hypothesize that the expression pattern of *Psc*RXLR1 could support a role in the initial infection process, possibly through dampening of host defense responses. Indeed, effectors from other oomycete plant pathogens, including *Pc*QNE from *Ps. cubensis* and members of the CRN family from *P. infestans*, have also been shown to elicit similar phenotypes when transiently expressed in *N. benthamiana* [3], [24], [47], supporting the classification of *Psc*RXLR1 as an effector protein with a putative role in virulence.

In the current study, ongoing analysis of the *Ps. cubensis* genome has expanded the candidate effector complement of *Ps. cubensis* to 271 sequences, revealing significant variation in the conserved translocation motif. While previous analyses revealed a near equal distribution of RXLR∶QXLR motifs in *Ps. cubensis*, our current work, based on a higher coverage draft genome sequence and predicted protein sequences rather than open reading frames (ORFs), predicts sequences with 20 different amino acid possibilities at the R1 position of the XXLR motif. Of these 20 predicted R1 substitutions, 19 have expression support (Table S1). While all 20 R1 substitutions have yet to be functionally validated, it is not surprising that *Ps. cubensis* effectors may in fact utilize a more diverse set of translocation motifs compared to the *Phytophthora* spp., given its obligate lifestyle and relatively narrow host range. Among *Phytophthora* spp., the conservation of the RXLR motif is well-established, yet there are additional classes of oomycete effectors, such as the CRN family, that appear to utilize disparate translocation motifs [34], [47]. Moreover, analysis of the *Py. ultimum* genome has identified an additional predicted translocation motif, YxSL[RK] [9]. Indeed, divergence of transport signal sequences is even more pronounced between oomycetes and the true fungi, which have no obvious conserved motifs that could function in transport and show high degrees of variation even within the same species [48]. For example, the effectors AvrM and AvrL567 from *Melampsora lini*, an obligate rust fungi with a similar lifestyle to *Ps. cubensis*, rely on unique N-terminal sequences for uptake [48]. These sequences, while different in regards to sequence similarity from the RXLR motif observed in *Phytophthora* spp., are similar in that they feature positively charged residues, implying that secondary protein structure may be a factor contributing to uptake of these proteins. Both *M. lini* and *Ps. cubensis* are obligate biotrophs with specific host ranges, which may have influenced the evolution of their effector repertoires to select for unique translocation motifs compared to those found in *Phytophthora* spp.

Preliminary analysis of the *Ps. cubensis* effector repertoire reveals minimal orthology with annotated effector proteins from *P. infestans*, similar to what has been observed when comparing the effector complement from *P. infestans* with *P. ramorum*, *P. sojae* or *H. arabidopsidis* [8], [27], [28]. Through extensive analysis using both evolutionary and comparative genomics, *Phytophthora* RXLR effector genes have been shown to be undergoing accelerated rates of birth and death evolution as well as both widespread gene duplication and loss events [7], [8]. As such, among the predicted RXLR effector genes from *P. infestans*, *P. sojae*, and *P. ramorum*, there are few genes with high degrees of sequence similarity or 1∶1∶1 orthology [7]. Similarly, the same phenomenon was observed in comparing candidate effector sequences from *Ps. cubensis* to those of *P. infestans*. Of 271 predicted *Ps. cubensis* sequences, less than half (41%) of these had significant similarity (e-value<1e^−5^) to predicted *P. infestans* proteins, and only 3 of these sequences had similarity to annotated *P. infestans* RXLR effector proteins. These results indicate that the effector repertoire *Ps. cubensis* utilizes to promote its virulence and pathogenicity on its hosts is, as could be predicted, very different than that utilized by *P. infestans*, and likely the other *Phytophthora* spp. as well. This is likely due to differing selective pressures on *Ps. cubensis* resulting from host specificity as well as differences in lifestyle between the two pathogens (i.e., obligate vs. non-obligate).

In this study, we identified minimal conservation between the predicted *Ps. cubensis* effector complement and effectors from *P. infestans*. We hypothesize that the identification and analysis of effector to non-effector relationships among oomycete plant pathogens is a valid measure to assess conservation and rates of evolution. Additionally, with the identification of *Psc*RXLR1, a splice variant of a non-effector gene, we posit that these types of analyses as well as a more thorough analysis of alternative splicing may provide a preliminary baseline to not only investigate evolutionary differences among oomycete plant pathogens, but to also infer the relationship between effector repertoire and the host-pathogen specificity and lifestyle. We have used several criteria (i.e., prediction of selection pressure, secretion, etc.) to identify and analyze the relationship between predicted *Ps. cubensis* effectors and their orthologs in *P. infestans*. As observed for other effector proteins, some *Ps. cubensis* effectors may have experienced stronger positive selection than most other proteins within the genome. Interestingly, in addition to varying significantly from the genome average, the distribution of ω for the *Psc*E-*Pi* pairs has two distinct peaks, representing groups of effectors under different levels of selection pressure. Thus, it appears that aside from acting as effectors during infection, some of these slower evolving genes may have additional, “housekeeping” roles that are yet to be uncovered. Despite computational evidence indicating that these slower evolving genes are likely effectors, their role(s) in pathogenesis remain to be established.

## Materials and Methods

### *Ps. cubensis* culture and growth conditions

*Ps. cubensis* was maintained on *Cucumis sativus* cv. ‘Vlaspik’ as previously described [24]. Cucumber plants were grown at 22°C with a 12 h light/dark photoperiod. For *Ps. cubensis* inoculation, sporangia were collected from heavily sporulating leaves by washing with cold sterile distilled water and collecting sporangia in a centrifuge tube. Sporangia were enumerated with a hemocytometer and suspended to a concentration of 1×10^5^ sporangia/ml in sterile distilled water. The underside of fully expanded 2^nd^ or 3^rd^ true leaves of 4-week-old cucumber plants were spray-inoculated, until run-off, with the suspension, and incubated for 24 h at 100% humidity in the dark. After 24 h, inoculated plants were moved to a growth chamber (22°C with a 12 h light/dark photoperiod).

### DNA and RNA Extraction

Genomic DNA of *Ps. cubensis* was isolated from sporangia of isolate MSU-1 using the DNeasy Plant Mini kit (Qiagen, Germantown, MD) with slight modifications. Sporangia were washed from heavily sporulating leaves with sterile distilled water and filtered through a 40 µm nylon cell strainer to remove residual plant debris. The resultant sporangia suspension was centrifuged, and the supernatant decanted. Sporangia were suspended in buffer AP1 containing RNase and 5 µl of Proteinase K and incubated at 37°C for 20 min. 50 µl of 425–600 µm acid washed beads were added to the sporangia suspension and sporangia disrupted for 3 min using a vortex. Subsequent DNA extraction steps were performed according to manufacturer's instructions.

*Ps. cubensis* total RNA was isolated as follows: sporangia were collected as described above for DNA isolation, yet re-suspended in buffer RLT from the RNeasy Plant Mini Kit (Qiagen, Germantown, MD) and disrupted as above. RNA isolation was performed according to the manufacturer's instructions. RNA samples were treated with DNase (Promega, Madison, WI) prior to use.

### Sequence, Assembly and Annotation of the *Ps. cubensis* Genome

Genomic DNA was isolated from *Ps. cubensis* MSU-1 as described above and libraries constructed using the Illumina Genomic DNA Sample kit (Illumina, San Diego, CA). Two separate paired-end libraries were end sequenced using an Illumina Genome Analyzer II (Illumina, San Diego, CA) at the UC-Davis Genome Center. The first library was sequenced with 84 bp reads and an insert size of 180 bp yielding 7.8 Gbp of sequence. The second library was sequenced with 100 bp reads and an insert size of 480 bp yielding 5.5 Gbp of sequence. Illumina reads were trimmed to 51 bp to remove low quality regions at the 3′ end of the reads. Reads with more than one N base or a base with a quality score less than 20 were removed. The reads were then searched against the Cucumber genome assembly [49] with Bowtie v0.12.7 [50] and matching reads were removed; 4.5 Gbp of sequence was retained following trimming and cleaning the reads. The trimmed and cleaned reads were assembled using Velvet v1.0.14 [25]. Three Velvet runs were performed with hash lengths of 31, 41, and 51 and coverage cutoffs of 7, 3.6, and 2, respectively. A minimum contig size cutoff of 200 bp was used for all the assembly runs. The contigs from each Velvet run were then merged into one assembly using the Minimus2 pipeline (http://sourceforge.net/apps/mediawiki/amos/index.php?title=Minimus2). Contaminant-containing and mitochondrial contigs were removed; the final assembly contains 35,546 contigs with an N50 contig size of 4.0 Kbp; the total assembly is 64.4 Mbp. Reads were deposited in the Sequence Read Archive at the National Center for Biotechnology Information under study number SRP011018. This Whole Genome Shotgun project has been deposited at DDBJ/EMBL/GenBank under the accession AHJF00000000. The version described in this paper is the first version, AHJF01000000.

The assembly was annotated using the MAKER [26] annotation pipeline. The FGENESH gene finder [51] was used with the *Phytophthora* matrix to produce the initial gene calls for the pipeline. All transcript and protein sequences from sequenced oomycete genomes were provided to MAKER to improve the quality of the annotation. In total, 23,519 loci and 23,522 gene models were predicted. Putative functional annotation was assigned by searching the gene models against UniRef100 using BLASTX (cutoff: 1E-5) and transferring the first hit with informative annotation.

### Identification and Cloning of *Psc*RXLR1, *Psc*_781.4 and PITG_17484

Amplification of the coding sequence of *Psc*RXLR1 was performed using DNA primers that correspond to the open reading frame of *Psc*RxLR1 (Figure 5). Subsequent isolation and cloning of *Psc*RxLR1 was performed by PCR using gene-specific primers and genomic DNA from *Ps. cubensis* sporangia. Resultant amplicons were cloned into the TA cloning vector pGEM-T-Easy (Promega), generating pGEM_*Psc*RXLR1. To ensure identification of a complete coding sequence, as well as to verify the absence of introns in the sequence, 3′ RACE (Rapid Amplification of cDNA Ends) was performed using the SMARTer RACE cDNA Amplification Kit (Clontech, Mountain View, CA). Amplification of *Psc_*781.4 was performed using 3′ RACE as described above and the final coding sequence was amplified using gene specific primers (Figure 5). Fidelity of all sequences was confirmed by DNA sequencing using the ABI 3730 Genetic Analyzer (Applied Biosystems, Foster City, CA).

*P. infestans* clone PITG_17484 was amplified from cDNA of *P. infestans* by PCR using gene-specific primers. Amplicons were subcloned into pGEM-T-Easy and sequences confirmed by sequencing.

### DNA cloning and construct preparation

To validate the predicted signal peptide (SP) of *Psc*RXLR1, the yeast signal trap assay was used [24], [32], [33]. A DNA fragment corresponding to the predicted 26 amino acid signal peptide (including start codon) was amplified by PCR using gene specific primers modified to include 5′ *Eco*R1 and 3′ *Xho*I restriction sites. Resultant amplicons were cloned into TA cloning vector pGEM-T-Easy (Promega), to yield pGEM-*Psc*RXLR1-SP. The plasmid pGEM-*Psc*RXLR1-SP was digested with *Eco*R1 and *Xho*I, the 84 bp SP fragment was gel purified, and ligated into the *Eco*R1/*Xho*I sites of the yeast signal trap vector pSUC2T7M13ROI [32], generating pSUC2-*Psc*RXLR1.

The SP sequence of PITG_17484 was similarly amplified and cloned, generating pSUC2-PITG_17484. Plasmids pSUC2T7M13ROI, pSUC2-*Psc*RXLR1 and pSUC2-PITG_17484 were transformed into the yeast *SUC2* minus strain YTK12 using the Frozen-EZ Yeast Transformation IITM kit (Zymo Research, Orange, CA) following the manufacturer's instructions. Transformants were selected on CMD-W plates (0.67% yeast nitrogen base without amino acids, 0.075% tryptophan dropout supplement, 2% sucrose, 0.1% glucose, and 2% agar) for 3 days at 28 °C. To confirm signal peptide function, YTK12 containing the pSUC2 constructs were grown on raffinose-containing YPRAA plates (1% yeast extract, 2% peptone, 2% raffinose, 2 µg/L Antimycin A, and 2% agar). Yeast strains were replicated onto YPDA plates (1% yeast, 2% peptone, 2% glucose, 0.003% adenine hemisulfate, and 2% agar) and CMD-W plates as equal viability controls. TYK12 without pSUC2 was used as a negative control. The detection of the secreted invertase activity with 2,3,5-triphenyltetrazolium chloride (TTC) was performed as described by Tian et al. [24].

For construction of plasmids used for localization and phenotype studies, the open reading frames of *Psc*RXLR1, *Psc*_781.4 and PITG_17484 (minus stop codons) were amplified and cloned into the Gateway entry vector pENTR/D-TOPO (Invitrogen, Carlsbad, CA), yielding pENTR-*Psc*RXLR1-GW, pENTR-*Psc*_781.4-GW, and pENTR-PITG_17484-GW, respectively. The destination vector pVKH18En6gw-cCFP [24] was used to create the C-terminal CFP fusions, using heterologous recombination *via* LR Clonase, as per the manufacturer's instructions (Invitrogen).

### Transient expression and localization in *N. benthamiana*

Infiltration and transient expression in *N. benthamiana* using *A. tumefaciens* was performed on 4–6 week old plants as described in Tian et al. [24]. *A. tumefaciens* strains were grown overnight at 28°C on Luria-Bertani (LB) plates containing 50 µg/mL rifampicin and 25 µg/mL kanamycin. *A. tumefaciens* clones were re-suspended in induction buffer (10 mM MES, pH 5.6, 10 mM MgCl_2_, 150 µm acetosyringone) and incubated at room temperature, shaking in the dark, for 2 hours prior to infiltration. *A. tumefaciens* suspensions were infiltrated at a final concentration of OD_600_ = 0.8.

*A. tumefaciens*-mediated transient expression in *N. benthamiana* for localization of *Psc*RXLR1-CFP, *Psc*_781.4-CFP, and PITG_17484-CFP with *At*PIP2A-YFP [38] was performed as described above. Visualization of fluorescently tagged proteins was observed using an Olympus Fluoview 1000 laser scanning confocal microscope. Images were adjusted for contrast in Canvas X (ACD Systems).

### Quantitative real time PCR

First strand cDNA was synthesized from 1 µg total RNA using the first-strand cDNA synthesis kit (USB, Cleveland, OH). Quantitative RT-PCR was performed using a Mastercycler ep Realplex real-time PCR (Eppendorf, Hamburg, Germany) using HotStart SYBR Green qPCR Master Mix (2x) (USB), as previously described [52]. For amplification of *Psc*RXLR1 transcripts, gene specific primers were designed to amplify a 50 bp fragment (Forward: 5′-TGCGTAGCATCGCCAACCGA-3 and Reverse: 5′- TCTTGCCAGCTGCATCGCGA-3′). Primers specific for the *Ps. cubensis* internal transcribed spacer (ITS) region were used as an endogenous control. Cycle parameters were as follows: 95°C for 2 min, followed by 40 cycles of: 95°C (15 sec), 60°C (15 sec) and 72°C (45 sec). Fold expression was calculated based on expression in sporangia. Data were analyzed by two-way ANOVA using Prism 4 (GraphPad Software).

### Splice variant analysis

Primers spanning the region of intron 1 were used (Figure S4) to amplify RT-PCR products from SP, 4, and 8 dpi cDNA samples and resultant products were cloned into the TA cloning vector pGEM-T-Easy. Fidelity of all sequences was confirmed by DNA sequencing as described above.

### Ortholog identification and sequence analysis

Candidate effector proteins were identified from the predicted proteome of *Ps. cubensis* generated from the draft genome assembly using a modified RXLR effector identification pipeline [28]. Orthologs of *Psc*RXLR1 were identified in *P. infestans* (http://www.broadinstitute.org/), *P. sojae* (http://genome.jgi-psf.org), *P. ramorum* (http://genome.jgi-psf.org) and *Py. ultimum* (http://pythium.plantbiology.msu.edu) with BLAST [53]. Signal peptides were predicted using SignalP 3.0 ([54]; http://www.cbs.dtu.dk/services/SignalP/) and protein motifs identified using InterProScan ([55], http://www.ebi.ac.uk/Tools/pfa/iprscan/). Amino acid alignments were generated using ClustalW2 ([56], http://www.clustal.org), and resultant figures generated using BoxShade v3.21 (http://www.ch.embnet.org/software/BOX_form.html). *P. infestans* effector sequences from Haas *et al.* [7] were used for analyses and *P. infestans* protein models were obtained from the *Phytophthora infestans* Sequencing Project (http://www.broadinstitute.org/).

### Defining paralogs and orthologs and evolutionary rate estimates

Synonymous and non-synonymous substitution rates (*Ks* and *Ka*, respectively) were determined using the yn00 program in PAML [57]. Protein sequences were aligned first and “back-translated” to coding sequence alignments. Very few pairs had run errors (e.g., NAN in PAML output), and those with run errors were excluded. Sequence pairs that were too similar (*Ks*≤0.005) or too divergent (*Ks*>3) were also excluded from further analysis. For each *Ps. cubensis* or *P. infestans* effector protein, the closest paralogous genes were identified using within-species BLAST searches and used for rate calculation. Rates between putative orthologs were calculated as well. Putative orthologs were identified globally between *Ps. cubensis*, *P. infestans* or *P. ultimum* by first determining pairwise sequence similarities between all genes in these species. For each *Ps. cubensis* protein, X, a protein in a second species, Y, is considered an ortholog if the following two conditions are met: 1) X is the reciprocal best match of Y and 2) X is located in a syntenic block where Y is found. Syntenic regions were established using Multiple Collinearity Scan [58], with 1e^−5^ as an alignment significance threshold, match size ≥5, and average intergenic distance.

### Molecular phylogenetic analysis

The full-length protein sequences of *Psc*RXLR1 and its orthologs were aligned using default parameters with MUSCLE [59]. The multiple sequence alignment was used to infer phylogenetic relationships between *Psc*RXLR1 and its orthologs using the Maximum Likelihood method, based on the JTT matrix-based model [60] with MEGA5 [61]. Bootstrap values (based on 500 replicates) for each node are given if >25%.

## Supporting Information

Figure S1 **Signal peptide distribution among ortholog pairs.** (A) Distribution of secreted or non-secreted proteins in the *Pseudoperonospora cubensis* – *Phytophthora infestans* ortholog baseline dataset. (B) Distribution of *P. infestans* orthologs of *Ps. cubensis* effectors that are predicted to be secreted. *Psc*-sec = *Ps. cubensis* secreted protein. *Psc*-non = *Ps. cubensis* non-secreted protein. *Pi*-sec = *P. infestans* secreted protein. *Pi*-non = *P. infestans* non-secreted protein.
(TIF)

Figure S2 **Relationship between** ***Psc*** **RXLR1 and oomycete orthologs.** Alignment of *Psc*RXLR1, *Psc*_781.4, PITG_17484 (*P. infestans*), PYU_T005955 (*Py. ultimum*), *P. ramorum* 96701_0_3435, and *P. sojae* 156165 amino acid sequences were generated using ClustalW and represented with BoxShade. *Psc*RXLR1 signal peptide is boxed in blue. The RXLR or RXLR-like domains are boxed in red. The green boxes represent the EamA domains found in each protein sequence. Stop codons are represented by asterisks.
(PDF)

Figure S3 **Heterologous expression of** ***Psc*** **RXLR1 specifically results in cell death in** ***Nicotiana benthamiana*** **.** Infiltration of *Psc*RXLR1_CFP with or without the plasma membrane marker construct *At*PIP2A-YFP results in chlorosis and necrosis 4 days post-inoculation (dpi). Circles mark the infiltration zones, visible at 0 dpi. Infiltration with *At*PIP2A-YFP alone does not result in any observable phenotype in *N. benthamiana* leaves.
(TIF)

Figure S4 **Multiple sequence alignments of splice variant isoforms.** (A) Alignment representing non-spliced, *Psc*RXLR1 isoform. (B) Alignment representing spliced isoform indicative of *Psc*_781.4.
(PDF)

Figure S5 **Heterologous expression of** ***Psc*** **_781.4 in** ***Nicotiana benthamiana*** **.** Infiltration and expression of *Psc*_781.4 does not result in any observable phenotype in *N. benthamiana* leaves at 4 days post-inoculation (dpi). Circles mark the infiltration zones, visible at 9 dpi.
(TIF)

Table S1 **List of candidate effectors of** ***Pseudoperonospora cubensis*** **, their annotation, signal peptide prediction, RXLR-like motif, amino acid sequence, and expression.**
(XLSX)

Table S2 **List of** ***Pseudoperonospora cubensis*** **effector and** ***Phytophthora infestans*** **ortholog pairs.**
(XLSX)
