# Supplementary figures and images for: Alternative Splicing of a Multi-Drug Transporter from Pseudoperonospora cubensis Generates an RXLR Effector Protein That Elicits a Rapid Cell Death
Source: PLoS One. 2012 Apr 5;7(4):e34701. doi: 10.1371/journal.pone.0034701 (PMC3320632; doi:10.1371/journal.pone.0034701)

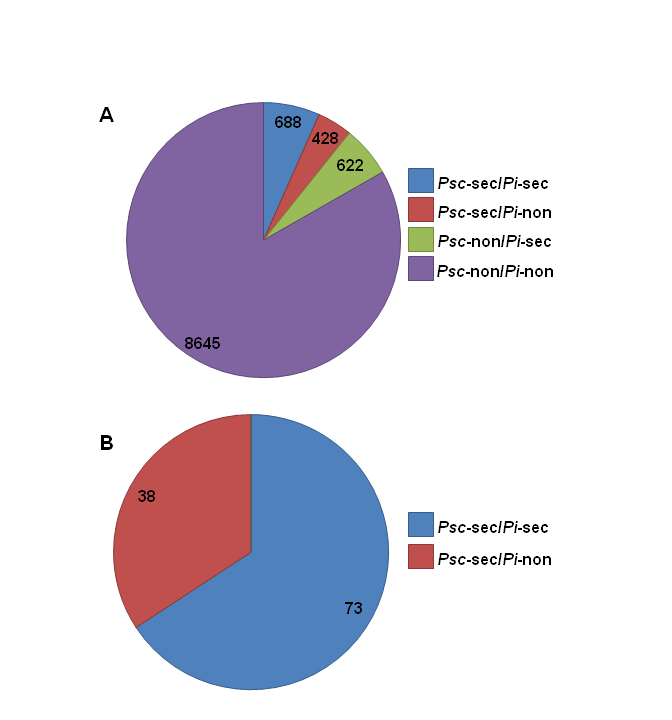

Supplement: Figure S1 — Signal peptide distribution among ortholog pairs. (A) Distribution of secreted or non-secreted proteins in the Pseudoperonospora cubensis – Phytophthora infestans ortholog baseline dataset. (B) Distribution of P. infestans orthologs of Ps. cubensis effectors that are predicted to be secreted. Psc-sec = Ps. cubensis secreted protein. Psc-non = Ps. cubensis non-secreted protein. Pi-sec = P. infestans secreted protein. Pi-non = P. infestans non-secreted protein. (TIF) [file pone.0034701.s001.tif]

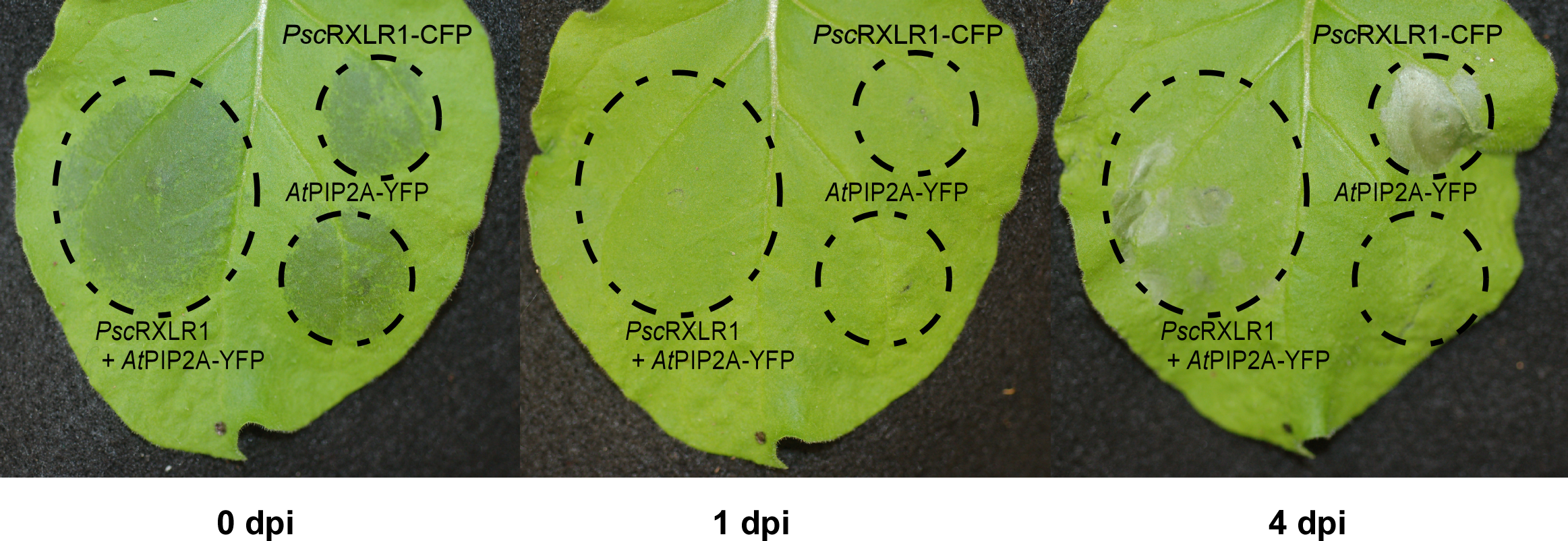

Supplement: Figure S3 — Heterologous expression of Psc RXLR1 specifically results in cell death in Nicotiana benthamiana . Infiltration of PscRXLR1_CFP with or without the plasma membrane marker construct AtPIP2A-YFP results in chlorosis and necrosis 4 days post-inoculation (dpi). Circles mark the infiltration zones, visible at 0 dpi. Infiltration with AtPIP2A-YFP alone does not result in any observable phenotype in N. benthamiana leaves. (TIF) [file pone.0034701.s003.tif]

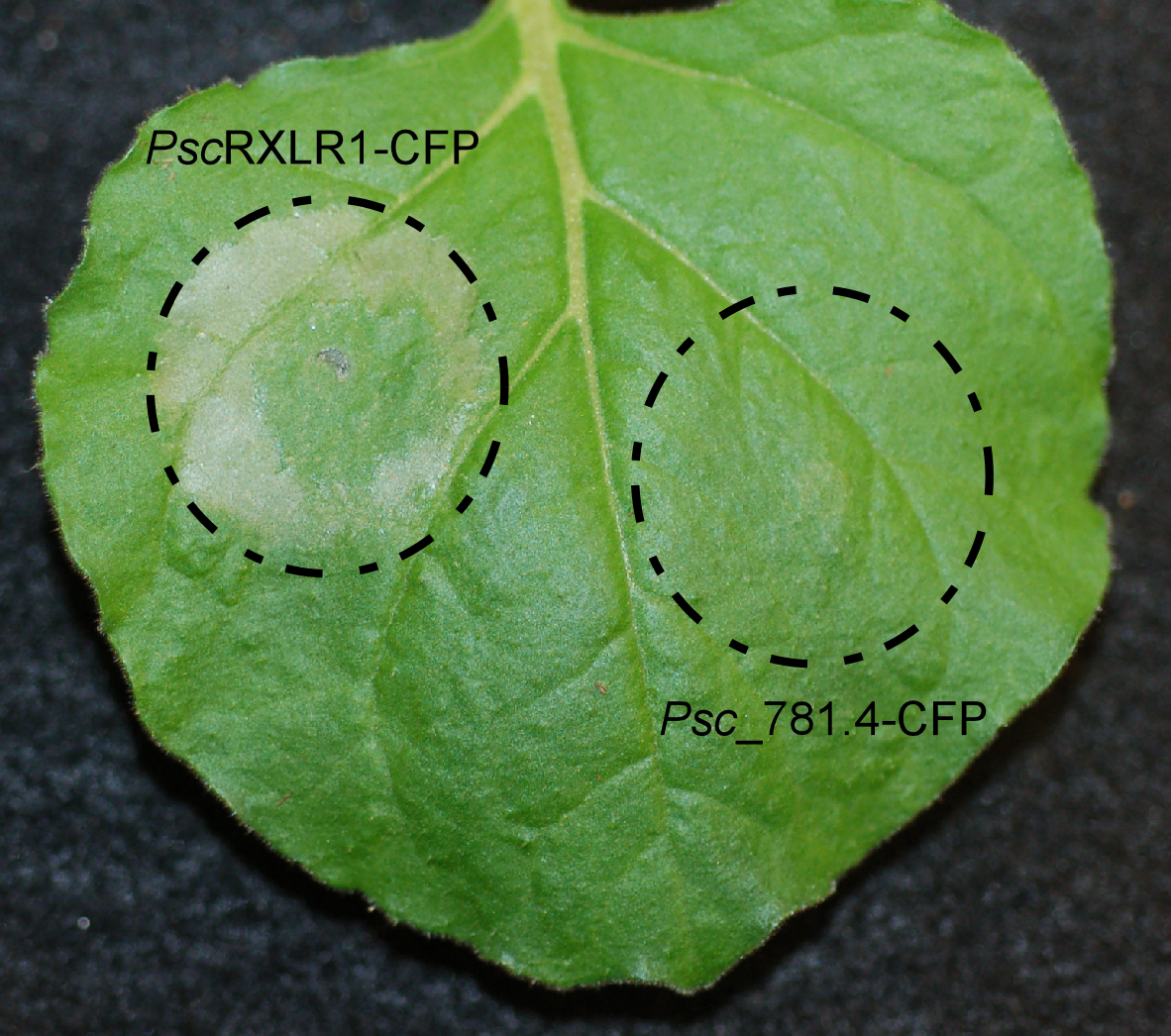

Supplement: Figure S5 — Heterologous expression of Psc _781.4 in Nicotiana benthamiana . Infiltration and expression of Psc_781.4 does not result in any observable phenotype in N. benthamiana leaves at 4 days post-inoculation (dpi). Circles mark the infiltration zones, visible at 9 dpi. (TIF) [file pone.0034701.s005.tif]
