# Supplementary material for: Alternative Splicing of a Multi-Drug Transporter from Pseudoperonospora cubensis Generates an RXLR Effector Protein That Elicits a Rapid Cell Death
Source: PLoS One. 2012 Apr 5;7(4):e34701. doi: 10.1371/journal.pone.0034701 (PMC3320632; doi:10.1371/journal.pone.0034701)
Supplement: Figure S2 — Relationship between Psc RXLR1 and oomycete orthologs. Alignment of PscRXLR1, Psc_781.4, PITG_17484 (P. infestans), PYU_T005955 (Py. ultimum), P. ramorum 96701_0_3435, and P. sojae 156165 amino acid sequences were generated using ClustalW and represented with BoxShade. PscRXLR1 signal peptide is boxed in blue. The RXLR or RXLR-like domains are boxed in red. The green boxes represent the EamA domains found in each protein sequence. Stop codons are represented by asterisks. (PDF) [file pone.0034701.s002.pdf]

```
PscRXLR1                 1   MVWLQLKKSGLGFTMSLSAVYGAVYAAAN-SVPAGKIDSGKKAMRHLENLPLLLASDSLE
Psc_781.4                1   ---------------MSLSAVYGAVYAAAN-SVPAGKIDSGKKAMRHLENLPLLLASDSLE
P. sojae 156165          1   ---------------MPLPAVYGAVYATATSSAPAGKIDSGKKAMRHLENLPLLVASDSLE
PITG_17484               1   ---------------MPLPAVYGAVYASATSSAPAG-----KKALRHLENLPLLVASDSLE
P. ramorum 96701_0_3435  1   ---------------MPLPAVYGAVYATATRSAPAGKIDSGKKAMHHLENLPLLIASDSLD
PYU1_T005955             1   ---------------MPAPAAVGIAVAAAA------NTDSGKKAMKQLEELPLLAVRHASD

PscRXLR1                 60  SVSTEGKWLPRFLRQAIMRSIANRIAGIILVSTSAFLASCIATLVKEDAVKLAPVEILFW
Psc_781.4                46  SVSTEGKWLPRFLRQAIMRSIANRIAGIILVSTSAFLASCIATLVKEDAVKLAPVEILFW
P. sojae 156165          47  SMSTDGKWLPRFMRRAVVRSVANRIAGLILVGTSAFLASCIATLVKDDAFKLSTVETLFW
PITG_17484               42  SMTTEGKWLPRFMRRAVVRSVANRIAGLILVGTSAFLASCITTLVKDDTIKLSAIEALFW
P. ramorum 96701_0_3435  47  SMSTEGKWLPRFMRRAVVRSVANRIAGLILVGTSAVLASCIATLTKDADFKLSPVETLFW
PYU1_T005955             41  AAIRTGSRLPKLVQKWYSKLSATQLEGVVLVAASAFTFSLLSTLIKYASQSMPSMETVFW

PscRXLR1                 120 RSLVSWLLTLVSS*-----------------------------------------------
Psc_781.4                106 RSLVSWLLTLVAITTTGVKTRLKKEYYRPIVLRSFTGCIATTLTIIMLQELAVSNAIAIT
P. sojae 156165          107 RSLVSWLLTLAAIAATGVKMRVKKEFQRPLLLRCFTGCIALTLTVLVIQKLEVSNATAIT
PITG_17484               102 RSLVSWFLTVAALATTSTKMRVKKEFNRPLTLRCVFGCISTTLTIGVLEKLAVSNATAVT
P. ramorum 96701_0_3435  107 RSLVSWLLTLAAIAATGIRMRVKKEFHRPLVLRCVTGCVAMTLTLLVLQTLAVSNATAIT
PYU1_T005955             101 RSFVAWLLNLVAVWQ----------------------------------MVLADASVLI

PscRXLR1                     ------------------------------------------------------------
Psc_781.4                166 YFSPLLAFAMAAKFLKEKPKLFAVACSVMCVIGAVLVVRPVFLFGKSGSTDASWYRRSMT
P. sojae 156165          167 YLSPLLAFAMAAFFLKEKPGAFTLACSALCVVGAVQVVRPAFVFGKNGSTDAKWYRRSMT
PITG_17484               162 YVSPLLAFAMATFFLKEKPGVFTAVCSALCVAGAVLVVRPAFLFGKSGSTDAKWYHRSMA
P. ramorum 96701_0_3435  167 YFSPLLAFAMAALFIKEKPDIFTVACSVVCVAGAILVVRPAFLFGKDGSTDAKWYRRSMA
PYU1_T005955             126 FTSPVMTFLLGAMVLKEKIDPVNMGYALFSFVGVICVVRPSFIFGNDHTTAG--------

PscRXLR1                     ------------------------------------------------------------
Psc_781.4                226 SFVTSYLFGESLAIGCAIIVVFMQAGAYVSLRSLQKVPHLVVMHYYLVTTTLVSLP*---
P. sojae 156165          227 SFVTSYLFGESLAIGCAVVTVVMQAGAYVSLRSLQKVPHLVVMNYFLLTMTLVSLIAILV
PITG_17484               222 SFVTSNLFGESLAVGCAVVVAFMQAGAYVSLRSLHKVEYLVVMQYYLFTMTLAALAAMLG
P. ramorum 96701_0_3435  227 SFVTSSLFGESLAIGCAAVVVFMQAGAYVSLRSLQKVPHLVVMHYFLLSMTLVSLVAVLV
PYU1_T005955             178 ------TDGSVFAIMCALLGAAAQAVAYVSMRRLQQVNYLVVINYFLLTSSVMSALSLLL

PscRXLR1                     ------------------------------------------------------------
Psc_781.4                    ------------------------------------------------------------
P. sojae 156165          287 VQHGKFKAGLSVETWGAILGTGALAFAEQLFLTRGFQFDGAGVLAATRLLHVGYEFVWGV
PITG_17484               282 IQHGKFKAGTSLETWGAIVGTGALAFVEQLFLTRGFQFDGAGVLAATRLLHVSCEFAWGV
P. ramorum96701_0_3435   287 VQHGKFKTDLSLGTWSAILGTGALAFAEQLFLTRGFQFDGAGVLAATRLLHVGCEFAWGV
PYU1_T005955             232 VQR-KFVIKMSLDVWLAVLGTGFLGFIGQLFLTRGFQLESAGTASVMRYLDVVFVFVWDI
```

```
PscRXLR1                             ------------------------------------------------------------
Psc_781.4                            ------------------------------------------------------------
P. sojae 156165                  347 ALLGTALNPWSASGAAATAAGVLFLALRRTARSREALAPHKNAYLH--------FSHARR
PITG_17484                       342 ILLGTALNPWSAGGAGVTAAGVLFLALRRVHTHWAARRSLRRILQ*--------------
P. ramorum 96701_0_3435          347 ILLGTALNPWSASGAAATAAGVLFLALRRVHTHWAARRSLRRMAAKPHKNAYLHFSSARR
PYU1_T005955                     291 TLLHERINAWSAVGALIICGSAIAIAIRKMQS*---------------------------

PscRXLR1                             ------------------------------------------------------------
Psc_781.4                            ------------------------------------------------------------
P. sojae 156165                  399 DELAAENPSWSVQQVSAELGRQWKALSAAERKPWVELAQFDKARFHTEAHHHV-NQQQSD
PITG_17484                           ------------------------------------------------------------
P. ramorum 96701_0_3435          407 EQLAEANPAWSVQQVSAELGRQWKSLAAVERKPWVELAQFDKARYHTEAHQHMRQQTDEQ
PYU1_T005955                         ------------------------------------------------------------

PscRXLR1                             ------------------------------------------------------------
Psc_781.4                            ------------------------------------------------------------
P. sojae 156165                  458 EQPEQAPPKRKKQSNEPRQPDTAYICFWKSQRPEVVAANPFLAAPLVSKEVGRQWRALSD
PITG_17484                           ------------------------------------------------------------
P. ramorum 96701_0_3435          467 PERPHLPTKRKKRPNEPRQPDTAYICFWKSRRPEVIAENPLLAAPSVSREEIARFQP---
PYU1_T005955                         ------------------------------------------------------------

PscRXLR1                             ------------------------------------------------------------
Psc_781.4                            ------------------------------------------------------------
P. sojae 156165                  518 DERQPTLAATTPDMLSALKTPLKDPFAPKPAKTAFQLFMSHNRESFMLLNMTINEFRAEM
PITG_17484                           ------------------------------------------------------------
P. ramorum 96701_0_3435          524 ------ALVATSEIPPALKAPLKDPFAPKPAKTGFQLFMSHNRESFTLLNMTINEFRTEM
PYU1_T005955                         ------------------------------------------------------------

PscRXLR1                             ------------------------------------------------------------
Psc_781.4                            ------------------------------------------------------------
P. sojae 156165                  578 SQLWKRLSDADKAEWHELAKEDQRRYDTEMNAYKPPAYMDLVVQRSHKRMEELRRLARED
PITG_17484                           ------------------------------------------------------------
P. ramorum 96701_0_3435          578 SQLWKRLSDADKNEWYELAKLDERRYETEMNAYKPPAYMESAVQRAHKRLDELRRLARRD
PYU1_T005955                         ------------------------------------------------------------

PscRXLR1                             ------------------------------------------------------------
Psc_781.4                            ------------------------------------------------------------
P. sojae 156165                  638 SAAPRLPMNAYNCYLSAKRQELVDRRPGRKNPEIMREIGVTWKALSDDERAVYQRKADED
PITG_17484                           ------------------------------------------------------------
P. ramorum 96701_0_3435          638 AAAPRLPMNAYNCYLSKERQELAVQRPDLKNPEIMREIGVTWKALSEDERASFQRKAEDD
PYU1_T005955                         ------------------------------------------------------------
```

```
PscRXLR1                     ------------------------------------------------------------
Psc_781.4                    ------------------------------------------------------------
P. sojae 156165          698 VERFRAEMEAHIAKKNEEEAANPLTKRRPRKRKEFDDEEELVKTPTVPRKKRKSGPPRRP
PITG_17484                   ------------------------------------------------------------
P. ramorum 96701_0_3435  698 VERFRADMEAYLTQQEEQRAVQEEQDVEPEVVREVVHEVVKEP--VVGRKKRKSVSPRRP
PYU1_T005955                 ------------------------------------------------------------

PscRXLR1                     ------------------------------------------------------------
Psc_781.4                    ------------------------------------------------------------
P. sojae 156165          758 KTAYNLMYMSKRTELLSTYQMSHNECSALCGKLWRQMSEAEREPYKRMAAEDKHRYEAEL
PITG_17484                   ------------------------------------------------------------
P. ramorum 96701_0_3435  756 KTAYNLMYMSKRAELLSTYQMSHNECSALCGRLWRQMSEEEREPYKRMAAEDKRRYETEM
PYU1_T005955                 ------------------------------------------------------------

PscRXLR1                     ------------------------------------------------------------
Psc_781.4                    ------------------------------------------------------------
P. sojae 156165          818 QVYNAQQEEANNKTLRDSAGFRHFLEAKRRENEAISSDEAAAIWQEMTEPHQLLWTELAR
PITG_17484                   ------------------------------------------------------------
P. ramorum 96701_0_3435  816 EIYNAEVDAANKKTLRESAGFSYFLEAKRRENEQISEGEAAAIWRDMLAPHQMLWTELAS
PYU1_T005955                 ------------------------------------------------------------

PscRXLR1                     ------------------------------------------------------------
Psc_781.4                    ------------------------------------------------------------
P. sojae 156165          878 DNKHKTSVERTAVDVLDTLL*---------------------------------------
PITG_17484                   ------------------------------------------------------------
P. ramorum 96701_0_3435  876 DTPAPASSNATHQKGQTGMNGGRWTEQEHQSFLAGLRLYGREWKKVAAKIKTRTSAQIRS
PYU1_T005955                 ------------------------------------------------------------

PscRXLR1                     ------------------------------------------------------------
Psc_781.4                    ------------------------------------------------------------
P. sojae 156165              ------------------------------------------------------------
PITG_17484                   ------------------------------------------------------------
P. ramorum 96701_0_3435  936 HAQKYFAKLARDDEMRKHSGLSMIMAGSIGYFSDGGSSVAQNSGDDDAEASDASRQMARA
PYU1_T005955                 ------------------------------------------------------------

PscRXLR1                     ------------------------------------------------------------
Psc_781.4                    ------------------------------------------------------------
P. sojae 156165              ------------------------------------------------------------
PITG_17484                   ------------------------------------------------------------
P. ramorum 96701_0_3435  996 RSAGQSKGTAAILIAPMGSAVSGLYKQTTGATKKRARAAVTGFDGQLEMGAATSSFPYKL
PYU1_T005955                 ------------------------------------------------------------
```

```
PscRXLR1                           ------------------------------------------------------------
Psc_781.4                          ------------------------------------------------------------
Psojae_156165                      ------------------------------------------------------------
PITG_17484                         ------------------------------------------------------------
P. ramorum 96701_0_3435            QKRQNDATRVEYLPSQEELLAKASPNLRHRLSSLIEAELCALQVLSCYAMLQQQEQISAP
PYU1_T005955                       ------------------------------------------------------------

PscRXLR1                           ---------------------------------
Psc_781.4                          ---------------------------------
Psojae_156165                      ---------------------------------
PITG_17484                         ---------------------------------
P. ramorum 96701_0_3435       1116 RQKTKRQGSAKASTLGLPMLSTEQMPPTSSIY*
PYU1_T005955                       ---------------------------------
```
