# Supplementary material for: Alternative Splicing of a Multi-Drug Transporter from Pseudoperonospora cubensis Generates an RXLR Effector Protein That Elicits a Rapid Cell Death
Source: PLoS One. 2012 Apr 5;7(4):e34701. doi: 10.1371/journal.pone.0034701 (PMC3320632; doi:10.1371/journal.pone.0034701)
Supplement: Figure S4 — Multiple sequence alignments of splice variant isoforms. (A) Alignment representing non-spliced, PscRXLR1 isoform. (B) Alignment representing spliced isoform indicative of Psc_781.4. (PDF) [file pone.0034701.s004.pdf]

A

```
781_4 exon 1(last 100 bp)          TGCCAGCTGC ATCGCGACGT TGGTGAAAGA GGACGCCGTT AAATTAGCAC
781_4 exon1 F primer               ---------- ---------- ---------- ---------- ----------
4 dpi #6-8                         ---------- ---------- ---------- ---------- ----------
8 dpi #4-12                        ---------- ---------- ---------- ---------- ----------
SP #5-9                            ---------- ---------- ---------- ---------- ----------
INTRON 1                           ---------- ---------- ---------- ---------- ----------
781_4 intron 1 span R primer       ---------- ---------- ---------- ---------- ----------
781_4 exon 2 (first 100 bp)        ---------- ---------- ---------- ---------- ----------

781_4 exon 1(last 100 bp)          CCGTGGAGAT TTTGTTTTGG CGCTCACTCG TGTCTTGGCT GCTAACGCTT
781_4 exon1 F primer               ---------- ------TTGG CGCTCACTCG TGTCTTG--- ----------
4 dpi #6-8                         ---------- ------TTGG CGCTCACTCG TGTCTTGGCT GCTAACGCTT
8 dpi #4-12                        ---------- ------TTGG CGCTCACTCG TGTCTTGGCT GCTAACGCTT
SP #5-9                            ---------- ------TTGG CGCTCACTCG TGTCTTGGCT GCTAACGCTT
INTRON 1                           ---------- ---------- ---------- ---------- ----------
781_4 intron 1 span R primer       ---------- ---------- ---------- ---------- ----------
781_4 exon 2 (first 100 bp)        ---------- ---------- ---------- ---------- ----------

781_4 exon 1(last 100 bp)          ---------- ---------- ---------- ---------- ----------
781_4 exon1 F primer               ---------- ---------- ---------- ---------- ----------
4 dpi #6-8                         GTAAGCTCTT GACCCACTGA TATTGTACGA TGATTGCCTA ACAAATTCTT
8 dpi #4-12                        GTAAGCTCTT GACCCACTGA TATTGTACGA TGATTGCCTA ACAAATTCTT
SP #5-9                            GTAAGCTCTT GACCCACTGA TATTGTACGA TGATTGCCTA ACAAATTCTT
INTRON 1                           GTAAGCTCTT GACCCACTGA TATTGTACGA TGATTGCCTA ACAAATTCTT
781_4 intron 1 span R primer       ---------- ---------- ---------- ---------- ----------
781_4 exon 2 (first 100 bp)        ---------- ---------- ---------- ---------- ----------

781_4 exon 1(last 100 bp)          ---------- ---------- ---------- ---------- ----------
781_4 exon1 F primer               ---------- ---------- ---------- ---------- ----------
4 dpi #6-8                         GGTGATTGAT TACAAGGTTG CAATCACGAC TACTGGCGTT AAG-------
8 dpi #4-12                        GGTGATTGAT TACAAGGTTG CAATCACGAC TACTGGCGTT AAG-------
SP #5-9                            GGTGATTGAT TACAAGGTTG CAATCACGAC TACTGGCGTT AAG-------
INTRON 1                           GGTGATTGAT TACAAG---- ---------- ---------- ----------
781_4 intron 1 span R primer       ---------- ---------G CAATCACGAC TACTGGCGTT AAG-------
781_4 exon 2 (first 100 bp)        ---------- ------GTTG CAATCACGAC TACTGGCGTT AAGACGCGCT

781_4 exon 1(last 100 bp)          ---------- ---------- ---------- ---------- ----------
781_4 exon1 F primer               ---------- ---------- ---------- ---------- ----------
4 dpi #6-8                         ---------- ---------- ---------- ---------- ----------
8 dpi #4-12                        ---------- ---------- ---------- ---------- ----------
SP #5-9                            ---------- ---------- ---------- ---------- ----------
INTRON 1                           ---------- ---------- ---------- ---------- ----------
781_4 intron 1 span R primer       ---------- ---------- ---------- ---------- ----------
781_4 exon 2 (first 100 bp)        TGAAGAAAGA GTATTATCGC CCAATCGTGC TTCGATCATT CACGGGTTGC

781_4 exon 1(last 100 bp)          ---------- ------
781_4 exon1 F primer               ---------- ------
4 dpi #6-8                         ---------- ------
8 dpi #4-12                        ---------- ------
SP #5-9                            ---------- ------
INTRON 1                           ---------- ------
781_4 intron 1 span R primer       ---------- ------
781_4 exon 2 (first 100 bp)        ATCGCCACGA CACTTA
```

B

```
781_4 exon1 (last 100 bp)          TGCCAGCTGC ATCGCGACGT TGGTGAAAGA GGACGCCGTT AAATTAGCAC
781_4 exon1 F primer               ---------- ---------- ---------- ---------- ----------
4 dpi #3-1                         ---------- ---------- ---------- ---------- ----------
8 dpi #4-7                         ---------- ---------- ---------- ---------- ----------
SP #2-3                            ---------- ---------- ---------- ---------- ----------
781_4 intron 1 span R primer       ---------- ---------- ---------- ---------- ----------
781_4 exon 2 (first 100 bp)        ---------- ---------- ---------- ---------- ----------

781_4 exon1 (last 100 bp)          CCGTGGAGAT TTTGTTTTGG CGCTCACTCG TGTCTTGGCT GCTAACGCTT
781_4 exon1 F primer               ---------- ------TTGG CGCTCACTCG TGTCTTG--- ----------
4 dpi #3-1                         ---------- ------TTGG CGCTCACTCG TGTCTTGGCT GCTAACGCTT
8 dpi #4-7                         ---------- ------TTGG CGCTCACTCG TGTCTTGGCT GCTAACGCTT
SP #2-3                            ---------- ------TTGG CGCTCACTCG TGTCTTGGCT GCTAACGCTT
781_4 intron 1 span R primer       ---------- ---------- ---------- ---------- ----------
781_4 exon 2 (first 100 bp)        ---------- ---------- ---------- ---------- ----------

781_4 exon1 (last 100 bp)          ---------- ---------- ---------- ---------- ----------
781_4 exon1 F primer               ---------- ---------- ---------- ---------- ----------
4 dpi #3-1                         GTTGCAATCA CGACTACTGG CGTTAAG--- ---------- ----------
8 dpi #4-7                         GTTGCAATCA CGACTACTGG CGTTAAG--- ---------- ----------
SP #2-3                            GTTGCAATCA CGACTACTGG CGTTAAG--- ---------- ----------
781_4 intron 1 span R primer       ---GCAATCA CGACTACTGG CGTTAAG--- ---------- ----------
781_4 exon 2 (first 100 bp)        GTTGCAATCA CGACTACTGG CGTTAAGACG CGCTTGAAGA AAGAGTATTA

781_4 exon1 (last 100 bp)          ---------- ---------- ---------- ---------- ----------
781_4 exon1 F primer               ---------- ---------- ---------- ---------- ----------
4 dpi #3-1                         ---------- ---------- ---------- ---------- ----------
8 dpi #4-7                         ---------- ---------- ---------- ---------- ----------
SP #2-3                            ---------- ---------- ---------- ---------- ----------
781_4 intron 1 span R primer       ---------- ---------- ---------- ---------- ----------
781_4 exon 2 (first 100 bp)        TCGCCCAATC GTGCTTCGAT CATTCACGGG TTGCATCGCC ACGACACTTA
```
